# An integrative bioinformatics analysis of microarray data for identifying hub genes as diagnostic biomarkers of preeclampsia

**DOI:** 10.1042/BSR20190187

**Published:** 2019-09-03

**Authors:** Keling Liu, Qingmei Fu, Yao Liu, Chenhong Wang

**Affiliations:** 1 Department of Gynaecology and Obstetrics, Shenzhen Hospital of Southern Medical University, Shenzhen 518000, China; 2 Department of Gynaecology and Obstetrics, People’s Hospital of Baoan District, Shenzhen 518101, China

**Keywords:** diagnosis, differentially expressed genes, GEO, preeclampsia, support vector machines

## Abstract

Preeclampsia (PE) is a disorder of pregnancy that is characterised by hypertension and a significant amount of proteinuria beginning after 20 weeks of pregnancy. It is closely associated with high maternal morbidity, mortality, maternal organ dysfunction or foetal growth restriction. Therefore, it is necessary to identify early and novel diagnostic biomarkers of PE. In the present study, we performed a multi-step integrative bioinformatics analysis of microarray data for identifying hub genes as diagnostic biomarkers of PE. With the help of gene expression profiles of the Gene Expression Omnibus (GEO) dataset GSE60438, a total of 268 dysregulated genes were identified including 131 up- and 137 down-regulated differentially expressed genes (DEGs). Gene Ontology (GO) and Kyoto Encyclopedia of Genes and Genomes (KEGG) pathway enrichment analyses of DEGs suggested that DEGs were significantly enriched in disease-related biological processes (BPs) such as hormone activity, immune response, steroid hormone biosynthesis, metabolic pathways, and other signalling pathways. Using the STRING database, we established a protein–protein interaction (PPI) network based on the above DEGs. Module analysis and identification of hub genes were performed to screen a total of 17 significant hub genes. The support vector machines (SVMs) model was used to predict the potential application of biomarkers in PE diagnosis with an area under the receiver operating characteristic (ROC) curve (AUC) of 0.958 in the training set and 0.834 in the test set, suggesting that this risk classifier has good discrimination between PE patients and control samples. Our results demonstrated that these 17 differentially expressed hub genes can be used as potential biomarkers for diagnosis of PE.

## Introduction

Preeclampsia (PE) is a disorder of pregnancy that is characterised by hypertension and a significant amount of proteinuria [1] beginning after 20 weeks of pregnancy [2]. It is closely associated with high maternal morbidity, mortality, maternal organ dysfunction or foetal growth restriction [3,4]. It is estimated that the incidence rate of PE is between 3 and 10% of all pregnancies [5]. There are various risk factors for PE such as obesity, prior hypertension, old age and gestational diabetes. Additionally, oxidative stress, immunologic intolerance and angiogenic imbalance are considered as the main causes [6,7]. Therefore, it is necessary to identify early, novel diagnostic biomarkers of PE.

Recently, with the help of gene microarray and RNA-sequencing technology, as well as public databases including Gene Expression Omnibus (GEO), gene expression studies related to human diseases have been reported. However, there have been no reports describing the expression profiles in PE. For example, using microarray, the gene expression profiles of placental tissue from five controls and five PE samples were analysed [8]. In this study, a total of 224 transcripts were significantly differentially expressed. Functional enrichment analysis suggested that the top three significantly enriched functional groups are immune response, inflammatory response and chemotaxis. Another study identified 38840 CpG sites with significant DNA methylation alterations in early-onset PE (EOPET) [9]. These results may be useful for DNA methylation-based non-invasive prenatal diagnosis. In addition, Meng et al. [10] used DNA microarrays to find 939 differentially expressed genes (DEGs) between PE and normal pregnancies. They found Notch, Wnt, NF-κB and transforming growth factor-β (TGF-β) signalling pathways were aberrantly regulated in PE. Long non-coding RNAs (lncRNAs) were also reported to play important roles in the pathogenesis of PE. He et al. [11] described the lncRNA profiles in six PE placentas and five normal pregnancy placentas using microarray. They found 738 lncRNAs that were differentially expressed.

However, most of these studies were only focussed on determining DEGs between PE and controls samples. The value of early diagnostic biomarkers needs to be explored and to be further investigated.

In the present study, we have performed a multi-step integrative bioinformatics analysis of microarray data for identifying hub genes as diagnostic biomarkers of PE. With the help of gene expression profiles of the GEO dataset GSE60438, a total of 268 dysregulated genes were identified including 131 up- and 137 down-regulated DEGs. Functional and pathway enrichment analyses of DEGs suggested that these DEGs were significantly enriched in disease-related biological processes (BPs) such as hormone activity, immune response, steroid hormone biosynthesis, metabolic pathways and other signalling pathways. Using the STRING database, we established a protein–protein interaction (PPI) network based on the above DEGs. Module analysis and identification of hub genes was performed to screen a total of 17 significant hub genes. The support vector machine (SVM) model was used to predict the potential application of biomarkers in PE diagnosis with area under the receiver operating characteristic (ROC) curve (AUC) of 0.958 in the training set and 0.834 in the test set. Our results demonstrated that these 17 differentially expressed hub genes can be used as potential biomarkers for the diagnosis of PE.

## Materials and methods

### Selection of the GEO dataset and data processing

The gene expression microarray dataset GSE60438 [12] was downloaded from the GEO database (http://www.ncbi.nlm.nih.gov/geo/). The GSE60438 series (GPL10558 platform, Illumina HumanHT-12 V4.0 expression beadchip) contained a total of 77 samples (35 PE and 42 normotensive controls). First, the probe symbols were converted into gene symbols. When multiple probes corresponded to one specific gene, the median expression was considered as its final expression level.

### Screening of DEGs between PE and controls

To screen DEGs between PE and controls samples, the *limma* package [13] in R was used with the cut-off criteria of fold-change > 1.2 and *P*-value <0.05.

### Functional and pathway enrichment analyses of DEGs

To explore the biological characteristics of these DEGs, we performed Gene Ontology (GO) and Kyoto Encyclopedia of Genes and Genomes (KEGG) pathway enrichment analyses. GO enrichment analysis was performed on DAVID (version 6.8) (https://david.ncifcrf.gov/) [14]. Significant results for molecular function (MF), BP and cellular component (CC) were determined with a *P*-value <0.05. KEGG pathway enrichment analysis was performed by KOBAS (version 3.0) (http://kobas.cbi.pku.edu.cn/) [15], a web server was used for identification of enriched pathways. A *P*-value <0.05 was considered statistically significant.

### PPI network construction and module analysis

To establish the PPI network, the STRING (version 10.5) (http://string-db.org/) [16] online database was used. The parameter was set as medium confidence >0.4. To visualise the PPI network, Cytoscape (version 3.6.1) software (http://www.cytoscape.org/) was used to draw their interactions. The MCODE plug-in of the Cytoscape software was used to explore significant modules (the parameters were set to default).

### Identification of hub genes from the PPI network

To select hub genes from the PPI network, the plug-in cytoHubba [17] of the Cytoscape software was used, which was computed by two methods, Maximum Neighbourhood Component (MNC) and Maximal Clique Centrality (MCC).

### Prediction and evaluation of diagnostic biomarkers of PE using the SVM model

To explore diagnostic biomarkers of PE, we used the above hub genes as candidates to find their diagnostic value based on SVMs [18]. The *kernlab* package in R was used for SVM analysis. In brief, half of the samples (PE = 18, controls = 20) were randomly distributed as the training set, which was used to build a model. The remaining data were used as the test set. An ROC curve analysis was applied to evaluate the specificity and sensitivity of the SVM prediction model. The AUC was computed to estimate the diagnostic accuracy of the classifier.

### Placental tissue of PE

The placental tissues were obtained from five patients undergoing PE from the Department of Gynaecology and Obstetrics in People’s Hospital of Baoan District. The normal placental tissues were obtained from five patients without PE. And the informed consents were obtained from all patients. The study was approved by the Human Ethics Committee of People’s Hospital of Baoan District.

### RNA extraction and quantitative real-time PCR

The total RNA from PE patients and normal people was extracted by TRIzol (Invitrogen) according to protocol. The concentration and purity of RNA was examined using NanoDrop2000 spectrophotometer (NanoDrop Technologies, Wilmington, DE, U.S.A.). One microgram RNA was reverse transcribed using reverse transcription cDNA kit (Thermo Fisher Scientific, Waltham, U.S.A.) for synthesis of cDNA (42°C for 60 min, 70°C for 5 min, 4°C preservation). SYBR Green PCR Master Mix (Roche, Basel, Switzerland) was used to conduct qPCR experiment using Opticon RT-PCR Detection System (ABI 7500, Life Technology, U.S.A.). The PCR cycle was as follows: pretreatment at 95°C for 10 min; followed by 40 cycles of 94°C for 15 s, 60°C for 1 min, finally at 60°C for 1 min and at 4°C for preservation. Comparative cycle threshold (ΔΔ*C*_t_) was employed to analyse the expression of mRNA. β-actin expression was used for normalisation. The primer sequences followed are as shown in Supplementary Table S6.

## Results

### Identification of DEGs between PE and control samples

**Figure 1:**
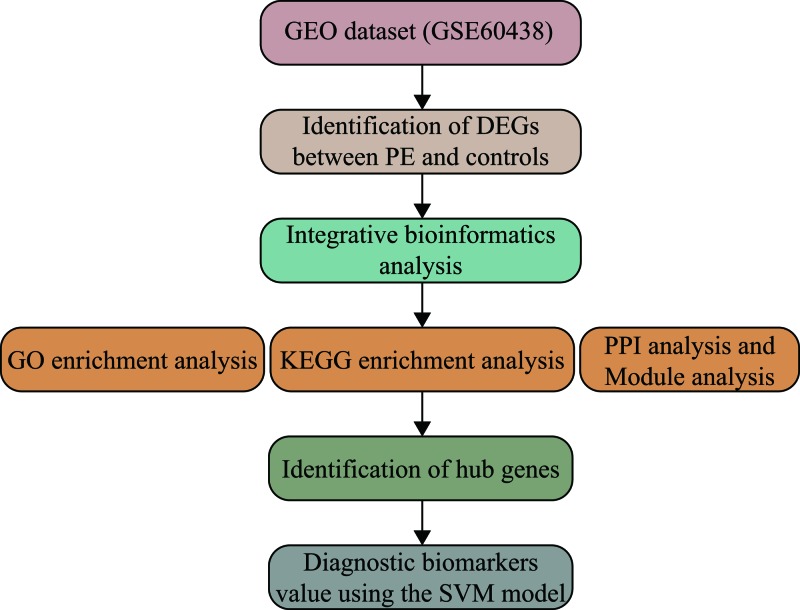
The workflow of the present study

**Figure 2:**
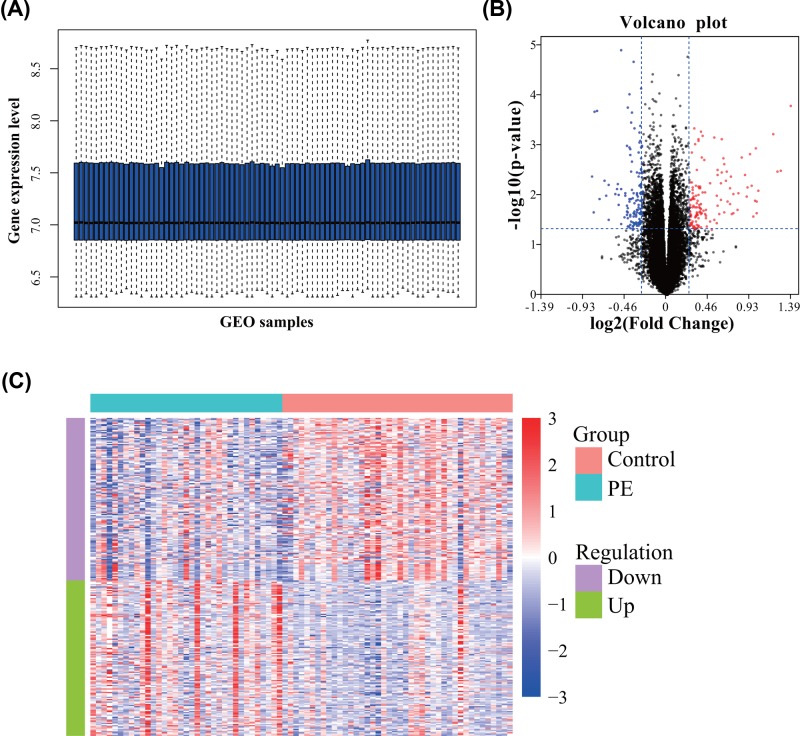
Identification of DEGs between PE and controls (**A**) The box plot of gene expression level in GSE60438. (**B**) The volcano plot of DEGs. (**C**) The heatmap of DEGs.

In the present study, we performed an integrative bioinformatics analysis of microarray data for identifying hub genes as diagnostic biomarkers of PE (Figure 1). First, we downloaded the gene expression profiles of GSE60438, which included a total of 77 samples (35 PE and 42 normotensive controls). The gene expression levels of all samples are shown in Figure 2A. After data processing, significant DEGs were screened using the *limma* package in R with a total of 268 dysregulated genes. Among them, 131 DEGs were up-regulated while 137 were down-regulated. The top ten up- and down-regulated DEGs in PE are shown in Table 1. The volcano plot and expression heatmap of DEGs are shown in Figure 2B,C.

**Table 1:** The top 10 up- and down-regulated DEGs

Genes symbol	log2-FC	*P*-value	Regulation
*CRH*	1.385139781	0.00016118	Up
*CGA*	1.26986941	0.003181303	Up
*KISS1*	1.236684519	0.003342022	Up
*CGB5*	1.188099886	0.000590546	Up
*PAGE4*	1.020501614	0.008133822	Up
*PSG3*	1.009911452	0.013572992	Up
*PSG4*	1.008965757	0.022509315	Up
*CGB8*	0.997798552	0.001124	Up
*PSG2*	0.988426217	0.012698631	Up
*PSG9*	0.968386595	0.026835911	Up
*GSTA1*	−0.828810367	0.004272094	Down
*FCN1*	−0.809162895	0.021796118	Down
*CCL2*	−0.797253724	0.00021502	Down
*CCL18*	−0.776742276	0.000202068	Down
*PI3*	−0.747174295	0.011841993	Down
*LTB*	−0.689241976	0.005131575	Down
*LYZ*	−0.673765329	0.010169589	Down
*CD48*	−0.648376667	0.006179909	Down
*AMICA1*	−0.647508338	0.031408632	Down
*SPP1*	−0.573396626	0.009359952	Down

### Integrative bioinformatics analysis of DEGs

**Figure 3:**
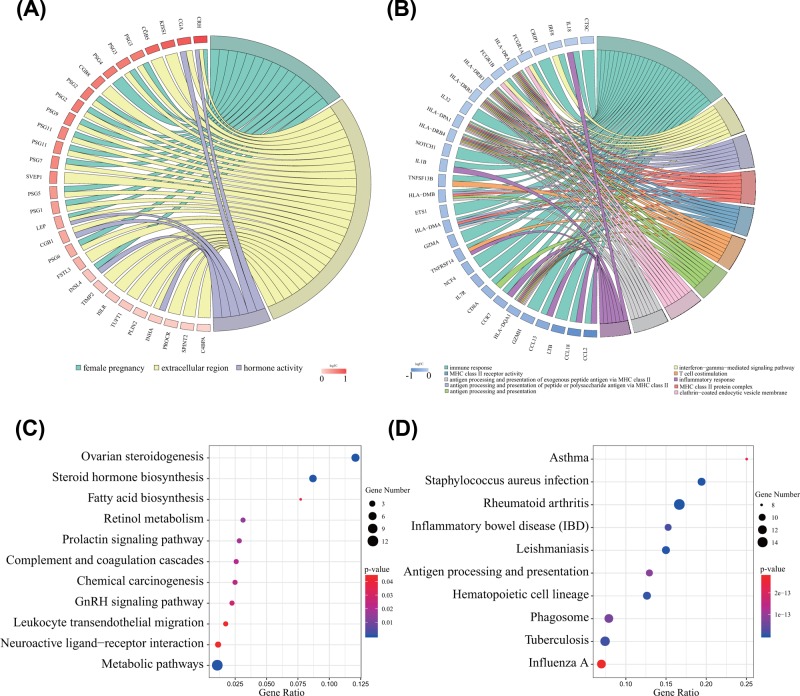
Functional and pathway enrichment analyses of DEGs (**A**) GO enrichment analyses of up-regulated DEGs. (**B**) GO enrichment analyses of down-regulated DEGs. (**C**) KEGG pathway enrichment analyses of up-regulated DEGs. (**D**) KEGG pathway enrichment analyses of down-regulated DEGs.

To explore the functional roles of the above DEGs, GO enrichment analysis of up- and down-regulated DEGs was first performed, respectively (Figure 3A,B and Supplementary Table S1). From the enrichment results of the CC category, we found that up-regulated DEGs were significantly enriched in the extracellular region, extracellular space and extracellular exosomes. In addition, DEGs associated with female pregnancy, androgen metabolic process, and negative regulation of glucagon secretion were enriched in the BP category. For the MF category, up-regulated DEGs were mainly enriched in hormone activity, TGF-β receptor binding and steroid hydroxylase activity. Moreover, down-regulated DEGs were enriched in MHC class II protein complex, clathrin-coated endocytic vesicle membrane and integral component of the lumenal side of endoplasmic reticulum membrane in the CC category. In the BP category, DEGs were mostly enriched in immune response, interferon-γ-mediated signalling pathway, and antigen processing and presentation of peptide or polysaccharide antigen via MHC class II. For the MF category, DEGs were enriched in MHC class II receptor activity, peptide antigen binding and receptor binding.

In addition, using the KOBAS analysis tool, the up-regulated DEGs were significantly enriched in ovarian steroidogenesis, steroid hormone biosynthesis and metabolic pathways (Figure 3C). However, the down-regulated DEGs were enriched in rheumatoid arthritis, *Staphylococcus aureus* infection and leishmaniasis (Figure 3D). The above results indicated that these DEGs are significantly enriched in disease-related BPs (Supplementary Table S2).

### Construction of the PPI network and module analysis

**Figure 4:**
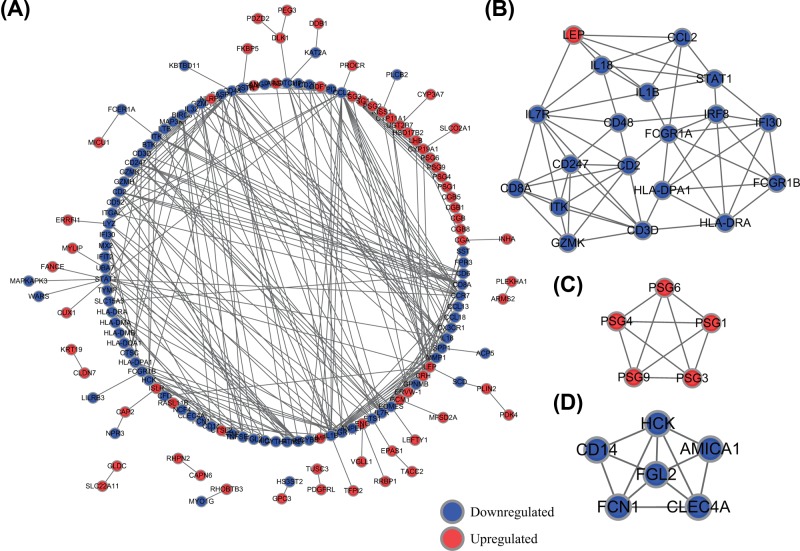
Construction of the PPI network and module analysis (**A**) Entire PPI network. (**B**) PPI network of module 1. (**C**) PPI network of module 2. (**D**) PPI network of module 3.

To explore the interactions between the above DEGs of PE, we performed PPI network analysis using the STRING database. The PPI network was constructed with 149 nodes and 358 edges including 67 up-regulated and 82 down-regulated genes (Figure 4A). Then, by using MCODE, we identified three key modules from the whole network (Figure 4B,C,D). DEGs in module 1 were significantly enriched in haematopoietic cell lineage, inflammatory bowel disease and Influenza A. However, DEGs of module 2 were not enriched in any pathways. DEGs in module 3 were significantly enriched in legionellosis, pathogenic *Escherichia coli* infection and pertussis (Supplementary Table S3).

To screen hub genes from the whole PPI network, we used the plug-in cytoHubba of the Cytoscape software, computed by MNC and MCC methods. We selected top 20 hub genes based on above two methods. Then, a total of 17 mutual hub genes were identified (Supplementary Table S4). They were *IL7R, IL18, CCL2, HLA-DRA, CD247, ITK, CD2, IRF8, CD48, GZMK, CCR7, HLA-DPA1, LEP, IL1B, CD8A, CD3D* and *GZMA*. Using KEGG pathway enrichment analysis, we found that above hub genes were significantly enriched in immune system, including Th17 cell differentiation, cytokine–cytokine receptor interaction, T-cell receptor signalling pathway and Primary immunodeficiency (Supplementary Figure S1 and Supplementary Table S5).

### The value of diagnostic biomarkers using the SVM model for PE

**Figure 5:**
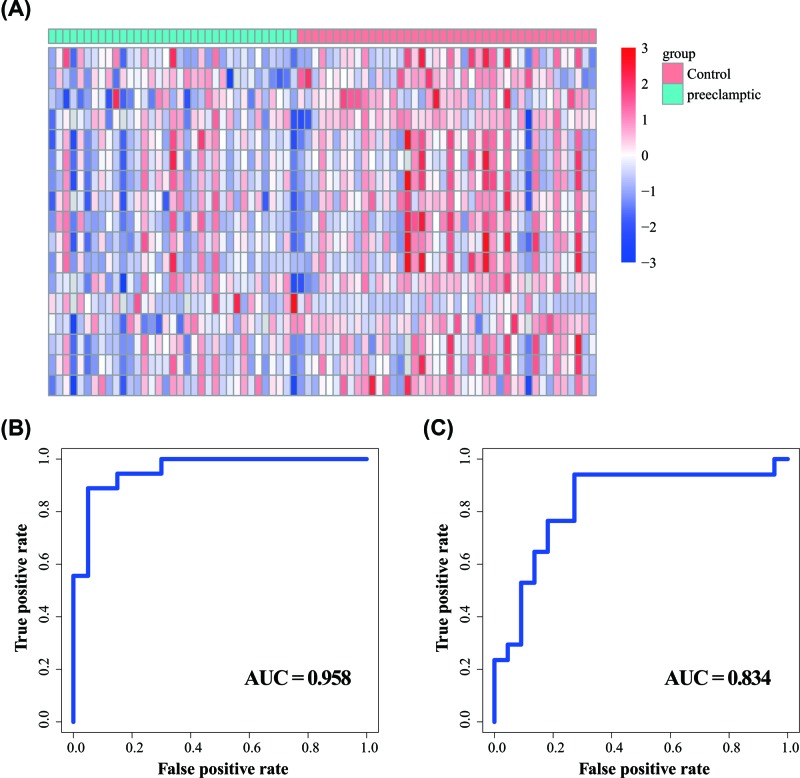
The value of diagnostic biomarkers using the SVM model for PE (**A**) The gene expression heatmap of all samples based on 17 hub genes. (**B**) ROC curves of SVM-based hub genes risk classifier in the training set. (**C**) ROC curves of SVM-based hub genes risk classifier in the test set.

To test the diagnostic value of these 17 hub genes of PE, we first performed the gene expression levels for all samples by heatmap. As shown in Figure 5A, there were some differentially expressed patterns between the PE and control samples. Then, we examined the diagnostic value of these 17 hub genes in PE using the SVM model. A risk classifier was developed based on gene expression levels, and the results suggested that the hub genes risk classifier had good discrimination between the PE and control samples with an AUC of 0.958 with the sensitivity of 95.0% and specificity of 66.7% (Figure 5B).

Then we validated the predictive value of these biomarkers in the PE test set. The ROC curves revealed that the sensitivity was 77.3%, the specificity was 76.5% and AUC was 0.834 for the test set (Figure 5C). These results suggested that the 17 differentially expressed hub genes can be used as potential biomarkers for the diagnosis of PE.

### Genes expression changes in PE

Next, in order to verify the results of previous bioinformatics analysis, the gene expression levels of *IL17R, CD8A, CD3D, CD48, CCL2* and *LEP* were detected by RT-qPCR between the placenta tissues of patients with PE and normal tissues. As shown in Supplementary Figure S2, compared with normal tissues, *IL17R, CD8A, CD3D, CD48* and *CCL2* mRNA expression levels were significantly down-regulated in the placenta of patients with PE, and *LEP* mRNA level was up-regulated, which was consistent with the results of bioinformatics analysis.

## Discussion

In the present study, we performed a multi-step integrative bioinformatics analysis of microarray data for identifying hub genes as diagnostic biomarkers of PE. With the help of gene expression profiles of the GSE60438 dataset, a total of 268 dysregulated genes were identified including 131 up- and 137 down-regulated DEGs. GO and KEGG pathway enrichment analyses of DEGs suggested that they were significantly enriched in disease-related BPs. PPI network construction, module analysis and identification of hub genes were performed to screen a total of 17 significant hub genes. The SVM model was used to predict the potential application of biomarkers in PE diagnosis with the AUC of 0.958 in the training set and 0.834 in the test set, suggesting that this risk classifier has good discrimination between PE patients and controls samples. Our results demonstrated that these 17 differentially expressed hub genes can be used as potential biomarkers for the diagnosis of PE.

Recently, there have been several gene expression profile studies about PE. Using the gene expression profiles of placental tissue from five controls and five PE patients, a total of 224 transcripts were found to be significantly differentially expressed [8]. In this study, they identified 91 genes that were up-regulated and 133 that were down-regulated in PE placentas compared with control placentas. Immune response, inflammatory response and chemotaxis were the top three significantly enriched functional groups. Using DNA microarrays, the gene expression profile of normal pregnancies (*n*=6) and patients with PE (*n*=6) was assessed [10]. A total of 939 genes including 483 up- and 456 down-regulated were identified that differed significantly in expression. IPA analysis revealed that MFs of these genes are involved in cellular function and maintenance, cellular development, cell signalling and lipid metabolism. Pathway analysis suggested that Notch, Wnt, NF-κB and TGF-β signalling pathways were aberrantly regulated in PE. He et al. [11] used six PE placentas and five normal pregnancy placentas to identify 738 lncRNAs that were differentially expressed (≥1.5-fold-change) among PE placentas compared with controls. They found that misregulation of LOC391533, LOC284100 and CEACAMP8 might contribute to the mechanism underlying PE. In another lncRNA study, Luo and Li [19] indicated that NR_027457 and AF085938 were significantly up-regulated, whereas G36948 and AK002210 were significantly down-regulated in PE using the qRT-PCR method. They also found NR_027457, AF085938, G36948 and AK002210 had potential diagnostic value for the detection of PE. In the present study, they identified potential diagnostic biomarkers in PE. Using the miRNA expression profile of GSE84260, a total of 65 differentially expressed miRNAs DEMIs including 32 up- miRNAs and 33 down-regulated miRNAs were identified [20]. In one review, the regulation and function of lncRNAs IGF2/H19, MEG3, SPRY4-IT1, HOTAIR, MALAT1, FLT1P1 and CEACAMP8 in placental trophoblasts was evaluated [21]. Moreover, the metabolic profiling was proved to have good clinical significance in the diagnosis of PE including PC (14:0/00), proline betaine and proline [22]. Although the above studies have found various DEGs between PE and normal samples, the value of early diagnostic biomarkers needs to explored and further investigated.

In our study, we have performed a multi-step integrative bioinformatics analysis to explore significant DEGs with diagnostic value for PE. Based on GO and KEGG pathway enrichment analyses, we found that these DEGs were significantly enriched in disease-related BPs. For example, up-regulated DEGs were mostly enriched in steroid hormone biosynthesis pathways. A previous study reported that the serum concentrations of steroid hormone, including oestrogen (E2) may be associated with PE [23]. The researchers found increased serum levels of asymmetric dimethylarginine (ADMA) in severe PE (sPE) may result from increased secretion from the placenta, and the increased progesterone/E2 ratio may play a role in the development of sPE by aggravating ADMA. In another study, it was reported that the levels of steroid hormones, particularly progesterone and E2, in the serum and placenta of women with PE are down-regulated, which may be mediated by the regulation of steroidogenic enzyme expression in the PE placenta [24]. We also found that down-regulated DEGs were enriched in various immune response processes. Immunological alterations and endothelial cell dysfunction have been widely accepted as major determinants for PE [25]. Different signalling pathways including chemokine, NF-κB, NOD-like receptor, T cell receptor, Toll-like receptor, TNF, MAPK and Notch signalling pathway were also found. MAPK/ERK phosphorylation is highly induced by inositols, which occurs in endothelial dysfunction in PE [26]. Moreover, dysregulation of Notch signalling components were observed in pregnancy disorders such as PE, which plays fundamental roles in different developmental processes of the placenta [27].

Based on our analysis, we identified a total of 17 hub genes with diagnostic value for PE. The concentrations of interleukin-18 (IL-18) were found to be significantly lower in the preeclamptic samples than in controls. Additionally, another study showed concentrations of IL-18 to be potentially linked to oxidative stress in PE [28]. However, serum IL-18 levels were significantly higher in patients with PE than in normal pregnant women in another study [29]. The CD247 molecule (CD247) was also proven to be a DEG in PE and considered as one of the top five DEGs with highest node degree in the PPI network [30]. Leptin (LEP) was differently expressed between the PE cases and controls. In addition, it might be related to the amount of placental pathology involved [31]. In one study, using the microarray dataset GSE44711, Ma et al. [32] found that granzyme A (GZMA) was a DEG between EOPET and normal controls. Additionally, they found that this gene was relevant to the immune system process, which may play significant roles in the progress of EOPET.

Based on the results of pathway enrichment analysis, hub genes were enriched in immune system. By summarising the clinical and experimental evidence, there was a contribution of the immune system to PE [33]. Immune-system alterations were associated with the origin of PE and the changes in T-cell subsets that may be seen in PE [34]. Other results demonstrated that immune imbalance can promote an inflammatory state during PE and it may be a potential therapeutic possibility for the management of PE [35]. Immune related genes such as HLA-*DRA, CD247, HLA-DPA1, IL1B* and *CD3D* were found as hub genes in our study. Above results suggested that these 17 hub genes may be potential therapeutic biomarkers of PE.

There are some limitations of the present study that should be acknowledged. Firstly, a sufficiently large number of clinical samples should be used to identify DEGs in PE. Secondly, further experimental verification such as qRT-PCR analysis should be performed.

## Conclusion

Our results demonstrated that these 17 differentially expressed hub genes can be used as potential biomarkers for the diagnosis of PE.

## Supporting information

**Supplementary Figure S1:** KEGG pathway enrichment analysis of 17 hub genes. (A) The dot plot of KEGG pathway enrichment results. (B) The connections of hub genes and KEGG pathways.

**Supplementary Figure S2:** Genes expression changes between PE and normal tissues.

**Supplementary Table S1:** Go functional enrichment analysis of DEGs.

**Supplementary Table S2:** KEGG pathway enrichment analysis of DEGs.

**Supplementary Table S3:** KEGG pathway enrichment analysis of each module.

**Supplementary Table S4:** The selection of hub genes based on MNC and MCC methods.

**Supplementary Table S5:** KEGG pathway enrichment analysis of 17 hub genes.

**Supplementary Table S6:** The primer sequences of IL17R, CD8A, CD3D, CD48, CCL2, LEP and β-actin.

## Abbreviations

ADMA: asymmetric dimethylarginine
AUC: area under the receiver operating characteristic curve
BP: biological process
CC: cellular component
DEG: differentially expressed gene
EOPET: early-onset PE
E2: oestrogen
GEO: Gene Expression Omnibus
GO: Gene Ontology
GZMA: granzyme A
IL-18: interleukin-18
KEGG: Kyoto Encyclopedia of Genes and Genomes
LEP: Leptin
lncRNA: long non-coding RNA
MCC: Maximal Clique Centrality
MF: molecular function
MNC: Maximum Neighbourhood Component
PE: preeclampsia
PPI: protein–protein interaction
ROC: receiver operating characteristic
SVM: support vector machine
TGF-β: transforming growth factor-β

## References

[1] Eiland E., Nzerue C. and Faulkner M. (2012) Preeclampsia 2012. J. Pregnancy 2012, 586578 10.1155/2012/586578 22848831 PMC3403177

[2] Al-Jameil N., Aziz Khan F., Fared Khan M. and Tabassum H. (2014) A brief overview of preeclampsia. J. Clin. Med. Res. 6, 1–7 24400024 10.4021/jocmr1682w PMC3881982

[3] Genest D.S., Falcao S., Gutkowska J. and Lavoie J.L. (2012) Impact of exercise training on preeclampsia: potential preventive mechanisms. Hypertension 60, 1104–1109 10.1161/HYPERTENSIONAHA.112.194050 23045469

[4] Say L., Chou D., Gemmill A., Tuncalp O., Moller A.B., Daniels J. (2014) Global causes of maternal death: a WHO systematic analysis. Lancet Glob. Health 2, e323–e333 10.1016/S2214-109X(14)70227-X 25103301

[5] Perry H., Khalil A. and Thilaganathan B. (2018) Preeclampsia and the cardiovascular system: an update. Trends Cardiovasc. Med. 10.1016/j.tcm.2018.04.009 29884568

[6] Powe C.E., Levine R.J. and Karumanchi S.A. (2011) Preeclampsia, a disease of the maternal endothelium: the role of antiangiogenic factors and implications for later cardiovascular disease. Circulation 123, 2856–2869 10.1161/CIRCULATIONAHA.109.853127 21690502 PMC3148781

[7] Lisowska M., Pietrucha T. and Sakowicz A. (2018) Preeclampsia and related cardiovascular risk: common genetic background. Curr. Hypertens. Rep. 20, 71 10.1007/s11906-018-0869-8 29971632 PMC6028827

[8] Song Y., Liu J., Huang S. and Zhang L. (2013) Analysis of differentially expressed genes in placental tissues of preeclampsia patients using microarray combined with the Connectivity Map database. Placenta 34, 1190–1195 10.1016/j.placenta.2013.09.013 24125805

[9] Blair J.D., Yuen R.K., Lim B.K., McFadden D.E., von Dadelszen P. and Robinson W.P. (2013) Widespread DNA hypomethylation at gene enhancer regions in placentas associated with early-onset pre-eclampsia. Mol. Hum. Reprod. 19, 697–708 10.1093/molehr/gat044 23770704 PMC3779005

[10] Meng T., Chen H., Sun M., Wang H., Zhao G. and Wang X. (2012) Identification of differential gene expression profiles in placentas from preeclamptic pregnancies versus normal pregnancies by DNA microarrays. OMICS 16, 301–311 10.1089/omi.2011.0066 22702245 PMC3369279

[11] He X., He Y., Xi B., Zheng J., Zeng X., Cai Q. (2013) LncRNAs expression in preeclampsia placenta reveals the potential role of LncRNAs contributing to preeclampsia pathogenesis. PLoS ONE 8, e81437 10.1371/journal.pone.0081437 24312300 PMC3842959

[12] Yong H.E., Melton P.E., Johnson M.P., Freed K.A., Kalionis B., Murthi P. (2015) Genome-wide transcriptome directed pathway analysis of maternal pre-eclampsia susceptibility genes. PLoS ONE 10, e0128230 10.1371/journal.pone.0128230 26010865 PMC4444079

[13] Ritchie M.E., Phipson B., Wu D., Hu Y., Law C.W., Shi W. (2015) limma powers differential expression analyses for RNA-sequencing and microarray studies. Nucleic Acids Res. 43, e47 10.1093/nar/gkv007 25605792 PMC4402510

[14] Huang da W., Sherman B.T. and Lempicki R.A. (2009) Systematic and integrative analysis of large gene lists using DAVID bioinformatics resources. Nat. Protoc. 4, 44–57 10.1038/nprot.2008.211 19131956

[15] Xie C., Mao X., Huang J., Ding Y., Wu J., Dong S. (2011) KOBAS 2.0: a web server for annotation and identification of enriched pathways and diseases. Nucleic Acids Res. 39, W316–W322 10.1093/nar/gkr483 21715386 PMC3125809

[16] Szklarczyk D., Morris J.H., Cook H., Kuhn M., Wyder S., Simonovic M. (2017) The STRING database in 2017: quality-controlled protein-protein association networks, made broadly accessible. Nucleic Acids Res. 45, D362–D368 10.1093/nar/gkw937 27924014 PMC5210637

[17] Chin C.H., Chen S.H., Wu H.H., Ho C.W., Ko M.T. and Lin C.Y. (2014) cytoHubba: identifying hub objects and sub-networks from complex interactome. BMC Systems Biol. 8, S11 10.1186/1752-0509-8-S4-S11 25521941 PMC4290687

[18] Furey T.S., Cristianini N., Duffy N., Bednarski D.W., Schummer M. and Haussler D. (2000) Support vector machine classification and validation of cancer tissue samples using microarray expression data. Bioinformatics 16, 906–914 10.1093/bioinformatics/16.10.906 11120680

[19] Luo X. and Li X. (2018) Long non-coding RNAs serve as diagnostic biomarkers of preeclampsia and modulate migration and invasiveness of trophoblast cells. Med. Sci. Monit. 24, 84–91 10.12659/MSM.907808 29302021 PMC5766055

[20] Luo S., Cao N., Tang Y. and Gu W. (2017) Identification of key microRNAs and genes in preeclampsia by bioinformatics analysis. PLoS ONE 12, e0178549 10.1371/journal.pone.0178549 28594854 PMC5464566

[21] Song X., Luo X., Gao Q., Wang Y., Gao Q. and Long W. (2017) Dysregulation of LncRNAs in placenta and pathogenesis of preeclampsia. Curr. Drug Targets 18, 1165–1170 10.2174/1389450118666170404160000 28382860

[22] Chen T., He P., Tan Y. and Xu D. (2017) Biomarker identification and pathway analysis of preeclampsia based on serum metabolomics. Biochem. Biophys. Res. Commun. 485, 119–125 10.1016/j.bbrc.2017.02.032 28188789

[23] Zheng J.J., Wang H.O., Huang M. and Zheng F.Y. (2016) Assessment of ADMA, estradiol, and progesterone in severe preeclampsia. Clin. Exp. Hypertens. 38, 347–351 10.3109/10641963.2015.1089880 27152507

[24] Shin Y.Y., Jeong J.S., Park M.N., Lee J.E., An S.M., Cho W.S. (2018) Regulation of steroid hormones in the placenta and serum of women with preeclampsia. Mol. Med. Rep. 17, 2681–2688 29207177 10.3892/mmr.2017.8165

[25] Steegers E.A., von Dadelszen P., Duvekot J.J. and Pijnenborg R. (2010) Pre-eclampsia. Lancet 376, 631–644 10.1016/S0140-6736(10)60279-6 20598363

[26] D’Oria R., Laviola L., Giorgino F., Unfer V., Bettocchi S. and Scioscia M. (2017) PKB/Akt and MAPK/ERK phosphorylation is highly induced by inositols: novel potential insights in endothelial dysfunction in preeclampsia. Pregnancy Hypertens. 10, 107–112 10.1016/j.preghy.2017.07.001 29153661

[27] Haider S., Pollheimer J. and Knofler M. (2017) Notch signalling in placental development and gestational diseases. Placenta 56, 65–72 10.1016/j.placenta.2017.01.117 28117145

[28] Roland L., Gagne A., Belanger M.C., Boutet M., Julien P. and Bilodeau J.F. (2010) Plasma interleukin-18 (IL-18) levels are correlated with antioxidant vitamin coenzyme Q(10) in preeclampsia. Acta Obstet. Gynecol. Scand. 89, 360–366 10.3109/00016340903576020 20199352

[29] Seol H.J., Lee E.S., Jung S.E., Jeong N.H., Lim J.E., Park S.H. (2009) Serum levels of YKL-40 and interleukin-18 and their relationship to disease severity in patients with preeclampsia. J. Reprod. Immunol. 79, 183–187 10.1016/j.jri.2008.10.003 19200605

[30] Song J., Li Y. and An R.F. (2015) Identification of early-onset preeclampsia-related genes and microRNAs by bioinformatics approaches. Reprod. Sci. 22, 954–963 10.1177/1933719115570898 25717061

[31] Kaartokallio T., Cervera A., Kyllonen A., Laivuori K., Kere J., Laivuori H.and FINNPEC Core Investigator Group (2015) Gene expression profiling of pre-eclamptic placentae by RNA sequencing. Sci. Rep. 5, 14107 10.1038/srep14107 26388242 PMC4585671

[32] Ma Y., Lin H., Zhang H., Song X. and Yang H. (2017) Identification of potential crucial genes associated with early-onset pre-eclampsia via a microarray analysis. J. Obstet. Gynaecol. Res. 43, 812–819 10.1111/jog.13275 28759171

[33] Jafri S. and Ormiston M.L. (2017) Immune regulation of systemic hypertension, pulmonary arterial hypertension, and preeclampsia: shared disease mechanisms and translational opportunities. Am. J. Physiol. Regul. Integr. Comp. Physiol. 313, R693–R705 10.1152/ajpregu.00259.2017 28978513

[34] Laresgoiti-Servitje E. (2013) A leading role for the immune system in the pathophysiology of preeclampsia. J. Leukoc. Biol. 94, 247–257 10.1189/jlb.1112603 23633414

[35] Ma Y., Ye Y., Zhang J., Ruan C.C. and Gao P.J. (2019) Immune imbalance is associated with the development of preeclampsia. Medicine (Baltimore) 98, e15080 10.1097/MD.0000000000015080 30946359 PMC6455976

