# Supplementary material for: An integrative bioinformatics analysis of microarray data for identifying hub genes as diagnostic biomarkers of preeclampsia
Source: Biosci Rep. 2019 Sep 3;39(9):BSR20190187. doi: 10.1042/BSR20190187 (PMC6722495; doi:10.1042/BSR20190187)
Supplement: Supplementary file 1 [file bsr20190187_Supp1.pdf]

Supplementary Information

Supplementary Table Legends

Supplementary Table 1. Go functional enrichment analysis of DEGs.

Supplementary Table 2. KEGG pathway enrichment analysis of DEGs.

Supplementary Table 3. KEGG pathway enrichment analysis of each module.

Supplementary Table 4. The selection of hub genes based on MNC and MCC methods.

Supplementary Table 5. KEGG pathway enrichment analysis of 17 hub genes.

Supplementary Table 6. The primer sequences of IL17R, CD8A, CD3D, CD48, CCL2, LEP and β-actin.

Supplementary Figure Legends

Supplementary Figure 1. KEGG pathway enrichment analysis of 17 hub genes. (A) The dot plot of KEGG pathway enrichment results. (B) The connections of hub genes and KEGG pathways.

Supplementary Figure 2. Genes expression changes between PE and normal tissues. IL17R, CD8A, CD3D, CD48 and CCL2 mRNA expression levels were significantly downregulated in the placenta of patients with PE, and LEP mRNA level was upregulated using RT-qPCR. N = 5/group, * p-value < 0.05, ** p-value < 0.01, *** p-value < 0.001 vs. NC and analyzed by Student’s t-test.

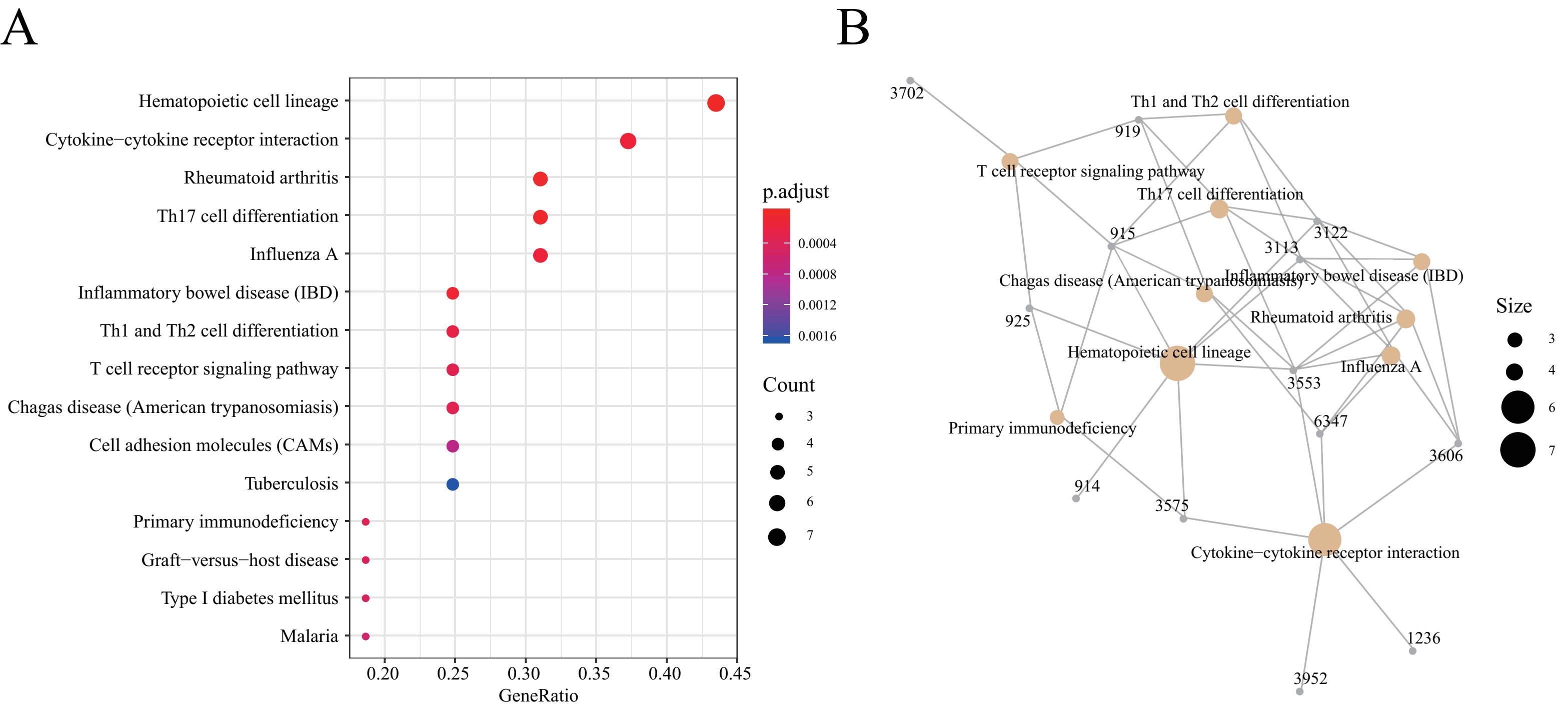

A
Hematopoietic cell lineage
Cytokine−cytokine receptor interaction
Rheumatoid arthritis
Th17 cell differentiation
Influenza A
Inflammatory bowel disease (IBD)
Th1 and Th2 cell differentiation
T cell receptor signaling pathway
Chagas disease (American trypanosomiasis)
Cell adhesion molecules (CAMs)
Tuberculosis
Primary immunodeficiency
Graft−versus−host disease
Type I diabetes mellitus
Malaria
0.20 0.25 0.30 0.35 0.40 0.45
GeneRatio
p.adjust
0.0004
0.0008
0.0012
0.0016
Count
3
4
5
6
7
B
3702
Th1 and Th2 cell differentiation
919
T cell receptor signaling pathway
Th17 cell differentiation
915
3122
3113
Chagas disease (American trypanosomiasis)
Inflammatory bowel disease (IBD)
925
Rheumatoid arthritis
Hematopoietic cell lineage
3553
Influenza A
6347
Primary immunodeficiency
3606
914
3575
Cytokine−cytokine receptor interaction
1236
3952
Size
3
4
6
7

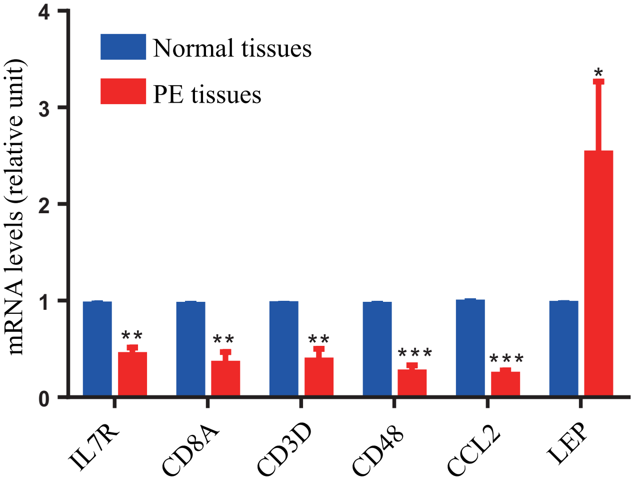

Normal tissues
PE tissues
mRNA levels (relative unit)
4
3
2
1
0
**
**
**
***
***
*
IL7R
CD8A
CD3D
CD48
CCL2
LEP
